# Diet quality indexes for use during pregnancy: a scoping review

**DOI:** 10.1093/nutrit/nuad138

**Published:** 2023-10-27

**Authors:** Liska Robb, Gina Joubert, Corinna May Walsh

**Affiliations:** Department of Nutrition and Dietetics, School of Health and Rehabilitation Sciences, Faculty of Health Sciences, University of the Free State, Bloemfontein, Republic of South Africa; Department of Biostatistics, School of Biomedical Sciences, Faculty of Health Sciences, University of the Free State, Bloemfontein, Republic of South Africa; Department of Nutrition and Dietetics, School of Health and Rehabilitation Sciences, Faculty of Health Sciences, University of the Free State, Bloemfontein, Republic of South Africa

**Keywords:** diet quality, diet quality index, first 1000 days, pregnancy, scoping review

## Abstract

**Aims:**

High diet quality is related to better health outcomes in general. During pregnancy, a high-quality diet is of paramount importance to promote optimal maternal and neonatal outcomes. This is a scoping review of research available on diet quality indexes (DQIs) for use during pregnancy that summarizes the DQIs in terms of development, country of origin, population used, components, scoring and weighting of components, and evaluation. Furthermore, the DQIs are discussed narratively to inform and direct the development of improved and country-specific DQIs for pregnancy.

**Methods:**

The EBSCOhost database was used to identify English-language, peer-reviewed articles published between 2000 and 2023, from which 11 publications were identified that describe the development of pregnancy-specific DQIs. This review followed the Preferred Reporting Items for Systematic Reviews and Meta-Analyses Extension for Scoping Reviews model.

**Results:**

Almost all DQIs (n = 9 of 11) were developed in high-income countries, using dietary intake data from food frequency questionnaires. Several DQIs (n = 5 of 11) used the US Healthy Eating Index as basis and modified it in various ways. Almost all DQIs included both foods and nutrients as components (n = 9 of 11), with vegetables being the most commonly included component alone (n = 8 of 11) or combined with fruit (n = 2 of 11).

**Conclusion:**

Because most DQIs were developed using dietary guidelines, recommendations, and dietary intake data from high-income countries, it is recommended that pregnancy-specific DQIs be developed and validated to reflect the nutrition guidelines for lower-income and culturally diverse countries.

## INTRODUCTION

The importance of adequate nutrient intake during pregnancy to promote optimal outcomes is well described.^1–3^ Because of increased nutrient needs, various cardiovascular, renal, endocrine, and metabolic adaptations occur to improve the absorption and use of nutrients in the body. However, these adaptations alone cannot ensure adequate nutrient status during pregnancy, even among women who have good nutritional status at the start of pregnancy.^4^ This results in a greater need for a high-quality diet during pregnancy to achieve the recommended nutrient intakes that are vital for positive pregnancy outcomes.^5^ New evidence suggests that single nutrients should not be investigated solely when determining the effect of diet on health outcomes, because humans consume food in different combinations and not as single nutrients. Foods also contain many biologically active components that are not defined as nutrients and may still affect health in numerous ways. Therefore, assessing overall diet quality is a useful approach for investigating diet-related health outcomes.^5^

The concept of “diet quality” has not been universally defined, although the inclusion of 3 aspects is generally accepted to contribute to better diet quality. These are (1) nutrient adequacy, which refers to the provision of appropriate energy and macro- and micronutrients to sustain health in the specific target group; (2) dietary diversity, which measures the intake of a variety of health-promoting foods and food groups, and (3) moderation, which refers to foods or nutrients that should be limited in the diet.^6^^,^^7^ Many diet quality indexes (DQIs) have been developed to quantify diet quality in different settings and populations.^7^^,^^8^ However, indexes developed specifically for use during pregnancy are less readily available.

Resource-limited populations in low-income countries often struggle to attain high-quality dietary intake because their diets often are based on cheaper, starchy foods, with relatively low intakes of nutrient-dense, animal-sourced foods, and fruits and vegetables.^9^ Similarly, pregnant women from high-income countries such as Canada and the United States may also not consume a high-quality diet.^10^^,^^11^ The additional nutrient requirements of pregnancy increase the risk of nutrient deficiencies as well as poor maternal health and pregnancy outcomes.

In view of the importance of a high-quality diet during pregnancy and the need to accurately measure diet quality in this population, a scoping review was conducted to systematically map the contents of the existing pregnancy-specific DQIs, as well as to identify possible gaps in knowledge. This information can contribute to informing and directing the development of improved and country-specific DQIs for pregnancy. The following research question was formulated: What is known from the literature regarding the availability and development of pregnancy-specific DQIs?

## METHODS

The review followed the Preferred Reporting Items for Systematic Reviews and Meta-Analyses Extension for Scoping Reviews model.^12^ Peer-reviewed journal articles were included if they were published between January 2000 and January 2023 and written in English. To be included in the review, articles had to describe the development of a pregnancy-specific DQI or had modified an established DQI to reflect country-specific dietary guidelines for pregnancy. Articles were excluded if they described the use of established DQIs that were only modified slightly (eg, 1 or 2 components were removed or replaced in an established index for use in a single study).

An electronic search of peer-reviewed literature was undertaken. EBSCOHost was used and included the following databases: Academic Search Ultimate; Africa-Wide Information; APA PsycArticles; APA PsycInfo; CAB Abstracts; CINAHL with Full Text; Health Source: Nursing/Academic Edition; Humanities Source Ultimate; MEDLINE; and Sociology Source Ultimate. An experienced university librarian drafted the search strategy, which was further refined during team discussions. The final search was conducted on January 30, 2023, using the following keywords: pregnan* and (“Healthy Eating Ind*” or “diet quality ind*”). Duplicates were removed by 1 reviewer (L.R.), after which all remaining titles, abstracts, and reference lists were screened for eligibility by 2 reviewers (L.R. and C.M.W.). Any disagreements about study selection and data extraction were resolved by consensus and discussion with other reviewers if needed.

A data-charting form was jointly developed by all authors to determine which variables to extract. One author (L.R.) independently charted the data, discussed the results, and continuously updated the data charting form in an iterative process in consultation with all authors. Any disagreements were resolved through discussion among all authors.

Data were extracted, summarized in tables, and were narratively discussed regarding the availability of pregnancy-specific DQIs, development, country of origin and population, components, scoring and weighting of components, and evaluation. The Health Sciences Research Ethics Committee of the University of the Free State granted ethics approval (approval UFS-HSD2018/0625/2603).

## RESULTS

**Figure 1:**
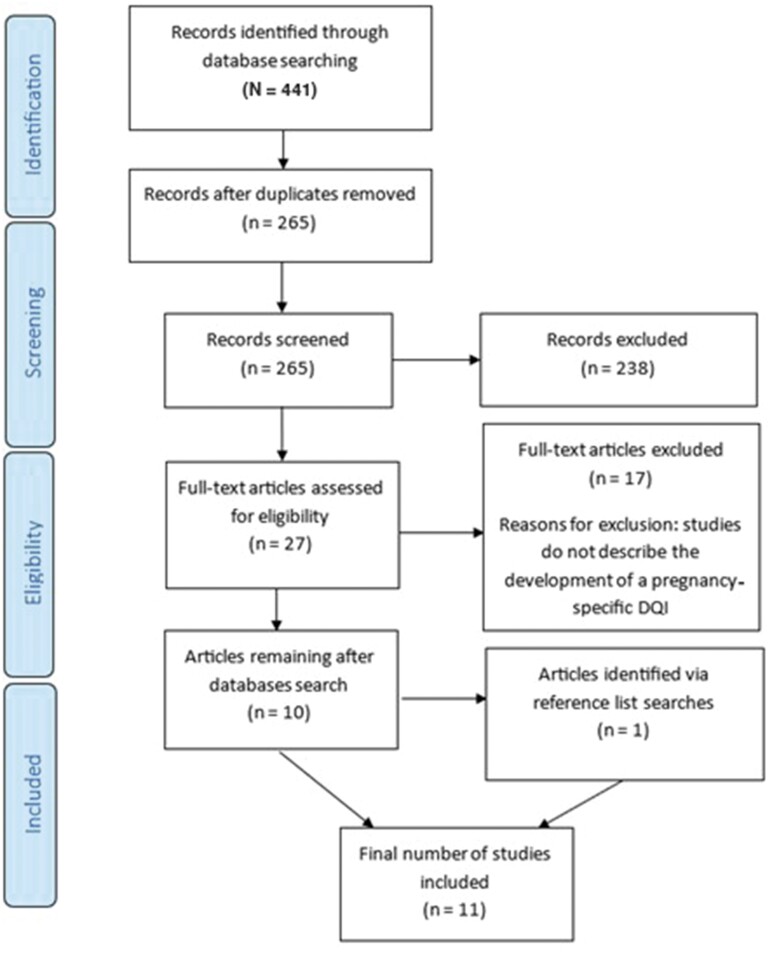
Flow chart representing screening and selection of studies. *Abbreviation:* DQI, diet quality index.

The search yielded 441 results. Numerous duplicate results were removed (n = 176). All remaining study titles and abstracts (n = 265) were read by 2 reviewers (L.R. and C.M.W.). After removing the articles that did not meet the inclusion criteria (n = 255), a total of 10 articles remained. One additional article was identified through screening of reference lists. Therefore, 11 articles are included in this review (Figure 1).

The following DQIs met inclusion criteria of this scoping review: Diet Quality Index for Pregnancy (DQIP),^13^ Alternate Healthy Eating Index for Pregnancy (AHEI-P),^14^ Mediterranean Diet Score–Pregnancy (MDS-P),^15^ Healthy Eating Index for Brazilian Pregnancy (HEIP-B),^16^ Diet Quality Index for Pregnancy (Canada) (DQI-Pc),^17^ Healthy Eating Index for pregnant women in Singapore (HEI-SGP),^18^ Dietary Assessment Tool (DAT),^19^ Healthy Food Intake Index (HFII),^20^ Probability of Adequate Nutrient Intake–Based Diet Quality Index (PANDiet),^21^ Diet Quality Index Adapted for Pregnant Women (IQDAG),^22^ and Prenatal Diet Quality Index (PDQI).^23^

Most of the 11 DQIs (n = 8) are based on already developed indexes, in turn either based on indexes for use in the general population (using the Healthy Eating Index [HEI]^24^ or the Brazil Healthy Eating Index Revised),^25^ or on indexes for use during pregnancy (using the DQIP, AHEI-P and HEIP-B) and further modified to reflect country-specific dietary guidelines and recommendations (Table S1 in the Supporting Information online).^3^^,^^13–23^^,^^25–45^

The DQI-P, MDS-P, and HFII are the only indexes with total original development (ie, they were not based on any existing DQI). In almost all articles (n = 9 of 11), dietary intake data used for scoring and validation purposes were collected using a food frequency questionnaire. The HFII is the only DQI developed specifically for use in women with an elevated risk of developing a disease condition during pregnancy (namely, gestational diabetes mellitus).

All DQIs originated in high-income countries, except for the HEIP-B and IQDAG, which were developed for the Brazilian population (Table S1). Sample sizes range from 318 to 27 529 pregnant women.

The number of components included in the DQIs ranged from 6 (DQI-Pc) to 13 (PDQI) (Table S2).^3^^,^^13–23^^,26,27,32,34,38,39,45–57^ However, the PANDiet includes only nutrients (n = 30 or 35 nutrients) and no other components. Most of the 11 DQIs (n = 9) include both food groups and nutrients as components, with 2 DQIs including additional components related to meal pattern (DQI-P and PDQI), 2 including the act of consuming an antenatal supplement (HEI-SGP and DAT), and 1 including a dietary diversity score (the PDQI). Various moderator components are included, and all DQIs contained at least 1 such component (eg, red meat, total fat, saturated fatty acids, trans fatty acids, ultraprocessed foods [UPFs], salt). Nutrient intake from antenatal supplements in addition to nutrients derived from foods (n = 3), as well as energy density (n = 3) are also included. Generally, fruit (n = 10) and vegetables (n = 10) (in different combinations), as well as micronutrients (iron, folate, and calcium) (n = 7) are the most commonly included components in the 11 DQIs.

Most components in all DQIs are scored as continuous variables, expressed as a percentage (0%–100%) of the adherence to the recommendations; however, some are scored in a categorical manner (Table S1). For the earlier DQIs, all components were weighted equally; however, since the development of the DQI-Pc in 2013, all subsequent DQIs contain components with different weightings, except the PANDiet, which weights all components equally. Three of the 11 DQIs consider energy intake for scoring purposes (DQIP, HEI-SGP, and IQDAG).

Various methods were used to evaluate the DQIs (Table S1). The methods that were most used include the associations between scores and nutrient and food group intakes, sociodemographic factors, and factors related to health and lifestyle, such as body mass index and smoking. For validation, 2 indexes (HFII and PDQI) included the evaluation of reproducibility of the index. The HFII also included the evaluation of component independence and construct validity.

## DISCUSSION

In the current scoping review, 11 primary articles that describe the development of pregnancy-specific DQIs published between January 2000 and January 2023 were identified. Dietary intake was mostly determined by using a food frequency questionnaire. Although this dietary intake assessment method is highly dependent on memory and the ability of the individual to estimate portion sizes, it can be used to determine macro- and micronutrient intake for up to several months. Dietary intake data used for scoring and validation purposes for the HEI-SGP were obtained from a single 24-hour dietary recall. Repeated 24-hour dietary recalls are more reliable than a single-day record,^58^ as applied in the PANDiet and IQDAG, which used 2 or 3 24-hour dietary recalls.

The HEI was used and modified in various ways for many of the 11 DQIs (n = 5). The HEI was developed by the US Department of Agriculture and measures the extent to which a diet aligns with recommendations made in the Dietary Guidelines for Americans. These guidelines are updated every 5 years. The first HEI was developed in 1995, and after 3 updates, the most current version is the HEI-2015.^24^ Using a DQI based on updated and well-researched nutrition guidelines is vital to obtain an accurate measurement of true diet quality. However, the applicability of the HEI in populations outside the United States remains to be determined.

Only 2 pregnancy-specific DQIs (HEIP-B and IQDAG) were created in a developing country (both in Brazil). Recently Bromage et al.^59^ developed a food-based diet quality score, the Global Diet Quality Score, using data from 10 African countries. The Global Diet Quality Score was associated with hemoglobin level, reduced anemia, and nutrient intake. The development of pregnancy-specific DQIs for use in developing countries should be prioritized to aid in the monitoring of diet quality and implementation of interventions to improve a poor diet quality in these populations.

Most of the DQIs included in this review (n = 9 or 11) include both food and nutrients as components. The PANDiet is unique because it is the only DQI that includes only nutrients as components (35 micro- and macronutrients as used for the French population, and 30 micro- and macronutrients as used for the US population). The authors state that advantages of nutrient-based DQIs include the large body of evidence that is available regarding recommended nutrient intake, as well as the ability for nutrient-based DQIs to be used in different countries and populations.^21^

Seven DQIs include a grains and cereal component. No DQI developed before 2015 includes whole grains as a component; however, 3 of the 5 more recently developed DQIs (ie, after 2015) include whole grains as a component (HEI-SGP, HFII, and PDQI). This finding may indicate an increased awareness of the health-promoting benefits of whole grains.^60^ However, a high intake of whole grains increases phytic acid intake, which may inhibit the intestinal absorption of important minerals such as iron and zinc. Poor iron and zinc absorption contributes to nutrient deficiencies, especially in low- and middle-income countries,^61^ which should be considered during DQI development and use in these countries. It is important to note that most of the indexes that do not include grains as a component (ie, AHEI-P, HEIP-B, and IQDAG) do include fiber as a separate component, which can serve as a proxy for grain intake.

All the indexes (excluding PANDiet) include fruits and vegetables, usually as separate components, confirming the consensus of the health benefits of these foods. Fruit intake during pregnancy improves cognitive development in children as measured at the age of 1 year, with lycopene and fructose being associated with neurodevelopment.^62^ The increased rate of energy metabolism during pregnancy increases the rate of free radical production, which can have detrimental effects on both the mother and neonate.^63^ Phytochemicals and micronutrients with antioxidant properties, abundant in fruit and vegetables, may be able to mitigate some of the detrimental effects of free radicals that could be present during pregnancy,^64^^,^^65^ but more research is needed to fully understand these interactions. In addition to these benefits, various researchers have found that intake of fruit and vegetables improved fetal growth, confirming the positive contribution of this food group toward diet quality during pregnancy.^66–68^ It is important to note that only 1 DQI (HEI-SGP) made a distinction between green leafy vegetables, yellow vegetables, and other types of vegetables. In general, green leafy vegetables and yellow fruit and vegetables are higher in a variety of nutrients and are associated with reduced risk of chronic diseases.^69^

Various protein foods are used as components in the current DQIs for pregnancy, of which the 3 most common components are beans, legumes, or pulses; dairy; and fish. These foods are not only rich in protein but contain high amounts of other vital micronutrients as well. In low- and middle-income countries, consumption of beans, legumes, or pulses, rather than expensive, animal-derived foods, may be a more feasible strategy for increasing protein intake.^70^ Intake of all meat is limited in 2 DQIs as an absolute amount (MDS-P and DAT) or as a ratio of white to red meat (AHEI-P and HEIP-B). Generally, most dietary guidelines warn against excessive intake of red meat, especially processed meat, for health and environmental reasons.^71–73^ Some authors do not agree with this practice and suggest that there is only weak observational evidence for the detrimental effect of red meat intake on health. They argue that limiting nutritious foods, such as meat, especially in vulnerable populations, can contribute to nutrient deficiencies.^74^^,^^75^ Because iron requirements are very high during pregnancy, and meat can contribute significantly to iron intake, limiting meat intake should be carefully weighed against the possible detrimental health effects of such a practice. However, a high intake of cheaper, processed meats in resource-poor settings may necessitate the inclusion of these meat products as a moderator component. Almost all DQIs for pregnancy (n = 7 of 11) include a separate component for iron intake to ensure adequate intake irrespective of the food or supplement source, highlighting its importance during pregnancy.

Components related to fats and fatty acids (excluding omega-3 fatty acids) are usually used as moderator components in the DQIs, meaning that consumption should be limited. Examples include limiting total fat, trans fatty acids, or saturated fatty acids. The IQDAG and PANDiet are the only DQIs that include omega-3 fatty acids as a component. A recent Cochrane review concluded that increased omega-3 fatty acid intake during pregnancy can reduce risks for premature birth and low birthweight infants.^76^ In 2015, in Sub-Saharan Africa, up to 500 000 neonates, according to estimates, died as a result of premature birth,^77^ highlighting the possible beneficial contribution of adequate omega-3 fatty acids to child survival.

The only micronutrient components that are included in most of the 11 DQIs (n = 7) are iron, calcium, and folate. However, the PANDiet includes 17 micronutrients as used for the US population and 20 micronutrients used for the French population. The HEI-SGP and DAT exclude individual micronutrients but include 1 component related to use of an antenatal supplement containing iron, folic acid, and calcium (HEI-SGP) and vitamin D, a multivitamin, and an omega-3 supplement (DAT). The use of such a component may be beneficial when accurate micronutrient intake data, such as that obtained from a quantified food frequency questionnaire or multiple 24-hour dietary recalls, are not available. However, if data regarding supplement use are not available, use of some DQIs might not be possible (namely, IQDAG, MDS-P, and PDQI) because they specifically include micronutrient intake from supplements in their score calculation. Two approaches are used in the DQIs to compare micronutrient intake to recommendations: using the recommended dietary allowance (RDA) (n =) or using the estimated average requirements (EAR) (n = 3) as reference values. According to the US Institute of Medicine (IOM), now the National Academy of Medicine, the RDA of a nutrient is the level at which the nutrient requirements of almost all particular individuals are met, whereas the EAR level would meet the requirements of half of the individuals in a specific group (because it constitutes the population mean). The IOM further states that the EAR can be used for both individuals (to examine the probability of inadequate intake of a specific pregnant woman) and groups (to examine the prevalence of inadequate intake in a population of pregnant women). The RDA, which is derived from the EAR (plus 2 standard deviations), should only be used in dietary intake assessment analysis for individuals.^78^ Comparing nutrient intakes to the RDAs is not a recommended practice when assessing nutrient intakes in populations.^79^

The bioavailability of iron influences recommended intake values. For nonpregnant individuals, the IOM-recommended intakes were determined assuming a bioavailability of 18.0% (a mixed Western diet); during pregnancy, a value of 25.0% was used.^80^ In 2001, the World Health Organization and Food and Agriculture Organization published recommended iron intakes at 5.0%, 10.0%, 12.0%, and 15.0% bioavailability for nonpregnant individuals. However, they refrained from making iron recommendations for pregnant women because “iron balance in pregnancy is highly dependent on iron stores.”^80^ The 2006 World Health Organization and Food and Agriculture Organization EAR value for iron intake during pregnancy at any level of bioavailability (>40 mg) is considerably higher than the EAR for pregnancy set by the IOM (22 mg); the IOM makes recommendations based on a typical North American diet, which has a higher iron bioavailability than that of many other populations (eg, traditional African diets). However, World Health Organization guidelines state that iron supplementation (30–60 mg/day) is required to meet the high demand of pregnancy and that pregnant women cannot solely rely on dietary modifications to meet iron requirements. A daily supplemental dose of 60 mg is preferred to a lower dose in areas with a prevalence of anemia in pregnant women of ≥ 40.0%.^81^ Thus, the prevalence of anemia in a country should also be considered when deciding on cutoff values for the iron (and possibly the red meat) component.

The intake of other micronutrients that are now known to be vital during pregnancy, such as choline, could be considered for inclusion in future DQIs. Choline, as a nutrient of concern during pregnancy, is notably absent from all DQIs. Given the importance of this micronutrient for fetal neurodevelopment^82^ and long-term metabolic health via epigenetic programming,^83^ it may add value to include the nutrient choline or choline-rich foods in pregnancy-specific DQIs.

Specific components used as moderators, including total fat, saturated fatty acids, trans fatty acids, UPFs, salt, added sugar, sugar-sweetened beverages, fast foods, and snacks, were included in some of the DQIs. In terms of the moderator components for fat intake, the HFII is the only DQI that specifically promotes types of foods instead of only focusing on nutrients (low-fat dairy and cheese, as well as plant-based cooking fats and spreads). The use of moderator components related to fat in DQIs reflects national and international recommendations for fat intake. In addition to potential cardiovascular concerns of a high-fat diet, it has been shown that a high-fat maternal diet can negatively influence the gut microbiome of the offspring.^84^ Chu et al^84^ recommend that more focus be placed on counselling related to maternal macronutrient intake to promote fat intake that is in line with current guidelines. UPFs are foods that are generally ready to consume with little or no preparation required^85^ and are usually high in sugar, salt, saturated fat, additives, and refined carbohydrates. The IQDAG is the only DQI that includes a moderator component for total UPF intake as a percentage of total dietary energy intake. Monteiro et al^86^ concluded that studies conducted in a variety of settings and applying various study designs consistently link UPFs to obesity, noncommunicable diseases, and death. In Brazil, Gomes et al^87^ investigated the effects of an educational intervention related to improved diet and activity with the aim of reducing UPF intake during pregnancy. The intervention was successful in significantly decreasing UPF intake in the intervention group.^87^ Findings such as these likely prompted the inclusion of UPFs in more recent DQIs. In the IQDAG, total UPF intake (as a percentage of total dietary energy intake) was defined according to the a new classification.^85^ However, specific cut points for total UPF intake were not available in Brazil to be implemented for scoring in the IQDAG; thus, the authors used the 16th and 85th percentiles of the distribution curve of UPF intake by the study population (equating to 18% to 45% of total energy intake) as cut points for scoring. In similar studies, UPF intake by pregnant women in Brazil ranged between approximately 15%^88^ and approximately 26%^87^ of total dietary energy intake depending on trimester. In St, Louis, Missouri, in the United States, pregnant women consumed a mean of 54.4% ± 13.2% of total dietary energy from UPF.^89^ It is evident that there is a wide range of intake of UPFs among pregnant women in different countries, which must be considered during the development of a DQI. The formulation of universal cut points for total UPF intake as a percentage of total energy can assist in better comparisons between countries.

Scoring of DQIs is characterized by challenges regarding weighting of components and interpretation of the overall score of all components. The systematic review of DQIs used in developing countries performed by Trijsburg et al^6^ recommends that all components should have equal weighting; however, if there is evidence that specific components are more important than others, different weightings may apply. The authors further state that there is currently no consensus regarding best practice for scoring in DQIs.^6^ In the present scoping review, the interpretation of the final scores of the DQIs for pregnancy we included is highly variable, which may make comparison between studies difficult. To overcome this, final scores could be converted to percentages and be interpreted as proposed by Basiotis et al^90^, with > 80.0% implying a good diet, 51.0%–80.0% implying a diet that needs improvement, and < 51.0% implying a poor diet. However, this method could be considered quite arbitrary because the components included and methods of scoring in the different DQIs will influence the final categorization. The differences in dietary assessment methods used in the DQIs could also contribute to variability between the studies.

Adapting scoring of DQIs to energy intake is recommended by some.^91^ Only 3 of 11 DQIs included in this review considered energy intake when calculating scores for different components. This is done to compensate for different recommended amounts of servings for food groups, based on individual energy requirements.

Validation of a DQI before widespread use is vital and should include the following steps^6^: assessment of reproducibility, reliability, relative validity, and construct validity; implementing a sensitivity and specificity analysis; and investigating associations between index and component scores with related health outcome(s). Most DQIs included in this review have not undergone these rigorous validation studies, which may limit their use. However, in general, the results of the primary validation processes in the different DQIs were as expected. For example, it was commonly found that DQI scores increased as dietary recommendations were met. Associations between most sociodemographic variables in the DQIs included in this review and scores also generally aligned with known results. For example, in a systematic review, Doyle et al^92^ found consistent positive associations among a higher prenatal diet quality and older age, higher educational attainment, and a higher income level, findings that are mostly similar to those of the present review. The HFII comprehensively described and implemented various validations techniques that can be used as a protocol for validation of other DQIs. Additionally, the use of biomarkers of exposure can be considered as a preferred reference method for validation of DQIs, as the errors associated with reporting dietary intake are mitigated.^6^ However, biomarkers (plasma folate and plasma folate, α- and β-carotene) were only used during the validation process of the HEI-SGP and the PANDiet included in the current review.

Consuming a high-quality diet during pregnancy can improve pregnancy outcomes. This concept should be measured and assessed periodically in populations to ascertain whether changes in diet quality are taking place and, if so, to identify the factors that contribute to these changes. This can inform policy and strategies that aim to improve maternal and child health.

Most pregnancy-specific DQIs included in this scoping review were developed using dietary guidelines, recommendations, and dietary intake data from high-income countries such as the United States, Canada, France, and Norway. Therefore, it is recommended that the development of such an index for use in developing countries be prioritized. Furthermore, because most DQIs included in this review had not undergone a rigorous validation process, the validation of DQIs before widespread use is recommended. It is further recommended that the use of biomarkers of exposure for validation is prioritized as part of this process. Validation using birth outcomes that are relatively simple to ascertain, such as gestation and birth weight, could prove beneficial, especially for use in low- and middle-income countries. Components that are not currently included in existing DQIs for pregnant women, but may be critically important to ensure high diet quality (eg, choline), should also be considered for inclusion in future DQIs.

An example of an initiative that was prioritized with the possibility of far-reaching advantages is The Global Diet Quality Project, which “aims to collect dietary quality data in the general adult population across countries worldwide, and to provide the tools for valid and feasible diet quality monitoring within countries. The project enables the collection of consistent, comparable dietary data across countries for the first time.”^93^ The development of a unifying method to measure diet quality during pregnancy should also be prioritized because the development of similar tools for pregnancy can provide the same advantages.

The exclusion of publications in languages other than English is acknowledged as a limitation of this review, especially considering DQIs that could have been developed in low- and middle-income countries. Furthermore, it is possible that unpublished, pregnancy-specific DQIs may be used in certain settings, but it was not attempted to identify and include these in this review. Finally, only articles that were published up to January 30, 2023, were included.

## CONCLUSION

Most DQIs for use during pregnancy were developed using dietary guidelines, recommendations, and dietary intake data from high-income countries. It is recommended that pregnancy-specific DQIs be developed and validated to reflect the dietary and nutrition guidelines for lower-income and culturally diverse countries.

## Supplementary Material

nuad138_Supplementary_Data

## Supporting Information

*Table S1*  **Development, scoring, and evaluation of DQIs for use in pregnancy**

*Table S2*  **Summary of components used in the current DQIs for pregnancy**
