# Supplementary material for: Diet quality indexes for use during pregnancy: a scoping review
Source: Nutr Rev. 2023 Oct 27;82(11):1622–30. doi: 10.1093/nutrit/nuad138 (PMC11464799; doi:10.1093/nutrit/nuad138)
Supplement: nuad138_Supplementary_Data [file nuad138_supplementary_data.zip › nuad138_Supplementary_Data/Table 1.pdf]

Table 1 Development, scoring and evaluation of DQIs for use in pregnancy

	Development				Scoring information		Evaluation
	**Country of origin**	**Population**	**Dietary intake assessment**	**Evidence base used for DQI development**	**Scoring and weighting of components**	**Interpretation of score**	
**Diet Quality Index for Pregnancy (DQIP)**<br>Bodnar & Siega-Riz (2002)[13]	North Carolina, United States of America	Mostly lower- to middle-income women from four antenatal clinics (N = 2063)	Dietary intake assessment took place during the second trimester of pregnancy utilising a 120-item Food Frequency Questionnaire (FFQ)	Dietary Guidelines for Americans (DGA)[26]; the Food Guide Pyramid (FGP)[27]; and the Institute of Medicine (IOM) Dietary Reference Intakes (DRIs)[3]	Each of the eight components contributes a maximum of 10 possible points<br>To assess adequacy of intake of the food groups and micronutrients, the percentage of the recommendation met as a continuous variable is calculated. This percentage is multiplied by 10 to determine a score for each of the food group components<br>Percentage of energy provided from fat as well as the meal pattern component were scored in a categorical manner with an optimal fat intake (≤30% of energy) and meal pattern (three meals and ≥ two snacks per day) obtaining the maximum score of 10 points<br>Equal weighting for all components	All component scores are summed to calculate a total diet quality score that ranges from 0 (worst) to 80 (best)	Nutrient intakes, food groups and sociodemographic factors<br>All nutrient and food group components (aligning with recommendations) showed a statistically significant association with an increased score<br>For selected micronutrient, protein, fibre, saturated fat and protein intakes, individuals with a high probability of having intakes that do not align with recommendations significantly decreased as scores increased<br>Women who were nulliparous, did not live in poverty, older and better educated had significantly higher scores
**Alternate Healthy Eating Index for Pregnancy (AHEI-P)**<br>Rifas-Shiman et al. (2009)[14]	Massachusetts, United States of America	Participants were enlisted at a multispecialty group practice in an urban area (N = 1777)	A self-administered questionnaire, which included a validated 166-item semi-quantitative FFQ, was used to determine dietary intake in women in their first trimester	The Healthy Eating Index (HEI)[28] developed by the USDA, further developed to the Alternate Healthy Eating Index (AHEI)[29] and subsequently modified for pregnancy	Each component contributes between 0 and 10 points. Intermediate intakes are scored proportionately between 0 and 10 by multiplying the number of servings that were consumed by 10, after which the score is divided by the standard for maximum score<br>Equal weighting for all components	All component scores are summed to calculate a total diet quality score ranging from 0 (worst) to 90 (best)	Pre-pregnancy BMI, pregnancy outcomes and sociodemographic factors<br>Participants who were older, leaner, nulliparous, and more educated had higher scores<br>The index appeared to be a predictor of at least two pregnancy outcomes, lower screening blood glucose level, and a slightly lower risk of developing preeclampsia

	Development				Scoring information		Evaluation
	Country of origin	Population	Dietary intake assessment	Evidence base used for DQI development	Scoring and weighting of components	Interpretation of score	
**Mediterranean Diet Score–Pregnancy (MDS-P)**<br><br>Mariscal-Arcas et al. (2009)[15]	Granada, Spain	Mother and son pairs who attended the San Cecilio University Hospital of Granada (N = 318) (equal rural/urban distribution)	A semi-quantitative FFQ was used for dietary intake assessment of pregnant women in their third trimester	The traditional Mediterranean diet as described by Trichopoulou et al.[30]	For food group and fatty acid components, 1 point is given if intake is above median intake of the group. Micronutrients score 1 point if intake is higher or equal to 67% of the recommended intake or if the woman is using a suitable antenatal supplement and 0 points if micronutrient intake is below this cut-off value<br><br>Equal weighting for all components	A score of ≤4 is categorised as poor compliance, 5–8 as adequate compliance, and ≥ 9 as high compliance	Sociodemographic factors, height, weight, body mass index, maternal weight change, weeks of gestation and newborn weight<br><br>A significant relationship was observed between lower scores and higher BMIs of the women at the start of the pregnancy and between higher scores and lower weights at the end of the pregnancies. A tendency was observed for an association between lower scores and shorter gestation periods
**Healthy Eating Index for Brazilian Pregnancy (HEIP-B)**<br><br>Melere et al. (2013)[16]	Porto Alegre and Bento Gonçalves, Southern Brazil	Pregnant women were (N = 712) selected at prenatal appointments at Primary Care Units and one Maternal-Infant Referral Centre)	A validated 88-item semi-qualitative FFQ was used to obtain dietary intake data information from women in the second and third trimester	The AHEI-P[14] was used and modified using the Brazilian Dietary Guide[31]	Each component contributes 0 to 10 points of the final score. Intermediate intakes are scored proportionately between 0 and 10 by multiplying the number of servings that were consumed by 10, after which the score is divided by the standard for maximum score<br><br>Equal weighting for all components	Good quality (> 80 points), needs improvement (51 – 80 points), and poor quality (< 51 points)	Nutrient intakes<br><br>Final scores showed moderate correlations with all nutrients, except for vitamin B12
**Diet Quality Index for Pregnancy (Canada) (DQI-Pc)**<br><br>Nash et al. (2013)[17]	Ontario, Canada	Women were enlisted from seven ultrasound clinics in London, Ontario (N = 2282)	Dietary intake assessment took place for food intake during the previous four weeks utilising a validated 106-item<br><br>FFQ	The DQI-P[13] was modified to reflect Canadian dietary guidelines[32]	Each component contributes 0 to 10 points of final score. Intermediate intakes are scored proportionately between 0 and 10 by multiplying the number of servings that were consumed by 10, after which the score is divided by the standard for maximum score<br><br>Each component is allocated a score of ten, except for the combined fruit and vegetable component that is scored to a maximum of 20 points	Six components are summed to provide a score out of 70, which is then converted to a score out of 100 points	Sociodemographic factors, nausea, exercise, smoking, depression, anxiety and social support<br><br>In the final parsimonious model, variables associated with higher scores were immigrants residing in Canada for <u><</u>5 years, <u>></u> 1 previous pregnancies, being married, more exercise, not

	Development				Scoring information		Evaluation
	Country of origin	Population	Dietary intake assessment	Evidence base used for DQI development	Scoring and weighting of components	Interpretation of score	
							smoking, lower anxiety levels, and greater social support from family
**Healthy Eating Index for pregnant women in Singapore (HEI-SGP)**<br><br>Han et al. (2015)[18]	Singapore, Republic of Singapore	Pregnant women receiving antenatal care in Kandang Kerbau Women's and Children's Hospital (KKH) and National University Hospital (NUH) were recruited (N = 955)	A 24-hour dietary recall and 3-day food diary were used to acquire dietary intake data form pregnant women in their second trimester	The index is adapted from the HEI[33] and Alternate Healthy Eating Index for Pregnancy (AHEI-P)[14] which was then modified to reflect the Singapore dietary guidelines for pregnant women[34]	Grains, fruit and vegetables, and meat and dairy, each score 20 points (as three subcategories). A maximum score is given if recommended intake levels are met and a score of zero if it is not met. Intermediate intakes are scored proportionately<br><br>For total fat and saturated fatty acids (SFA), a maximum score of 10 is given if intakes adhere to recommendations (30% energy for total fat, and 10% of energy for SFA), a zero score if it exceeds 40% of total energy intake for fat, and 20% of total energy intake for saturated fat. Intermediate ranges are scored proportionately<br><br>For antenatal supplements a score of 10 is given if their antenatal supplements contained all three of the important micronutrients (iron, folate and calcium), a score of 5 if they contain one or two of these micronutrients, and a score of 0 if they contain not one of these micronutrients	A raw score is calculated, ranging from 0–90. The raw score is then converted to a scale of 0–100	Sociodemographic factors, nutrient intakes, plasma folate, food groups, BMI, smoking, alcohol use and exercise<br><br>All nutrient and food group components as well as plasma folate (aligning with recommendations) showed a statistically significant association with an increased score<br><br>Pregnant women with higher scores were significantly older, had a lower BMI, were higher educated, had higher incomes, more likely to be married, nulliparous, and had not smoked before or during the pregnancy.
**Dietary Assessment Tool (DAT)**<br><br>Mullaney et al. (2016)[19]	Dublin, Ireland	Women attending the first antenatal visit at the Coombe Women and Infants University Hospital<br><br>(CWIUH) (N=524)	A semi-quantitative FFQ was used to collect dietary intake data from participants	Dietary intake guidelines for pregnancy published by various national and international health agencies as well as existing dietary quality indices such as the HEI-2005[28]	Each of the ten components was assigned an *a priori* weighting, based on their relevant nutritional importance during pregnancy. Each component yields a score which contributes to the final score (%). Weighting ranges from 5.4% (supplement use) to 12.5% for the fruit and vegetable component as well as the breakfast cereal component	Ten components are summed to provide a score out of 112, which is then converted to a score out of 100 points	Nutrient intakes<br><br>After micronutrient intakes were adjusted for total energy consumption, positive correlations were observed between scores and higher iron, folate, vitamin B12, calcium, magnesium, zinc, and iodine intakes<br><br>For energy and macronutrient intakes, negative correlation coefficients were observed between scores and total energy intake and percentage energy

	Development				Scoring information		Evaluation
	Country of origin	Population	Dietary intake assessment	Evidence base used for DQI development	Scoring and weighting of components	Interpretation of score	
							from fat, saturated fat, and non-milk extrinsic sugars
**Healthy Food Intake Index (HFII)**<br><br>Meinilä et al. (2016)[20]	Helsinki Metropolitan area and Lappeenranta, Finland	Participants were enlisted as part of the Finnish Gestational Diabetes Prevention Study (RADIEL) (N=727)	A semi-quantitative FFQ was used to collect dietary intake data from Finnish women at high-risk for gestational diabetes mellitus (GDM) due to obesity (BMI ≥30 kg/m^2), or a history of GDM, who were less than 20 weeks pregnant (n=492) or were planning pregnancy (n=235)	The components for the HFII reflect the recommendations of the food-based guidelines of the Nordic Nutrition Recommendations (NNR)[35]	Scores for dairy intake are assigned based on fat content. Scoring for cooking fat and fat spread components is based on SFA content. Food group cut-off values were established according to the dietary recommendation related to the number of servings per day. All other components (fast food, snacks, and sugar-sweetened beverages) are scored using cut-off values that are set as determined by calculating the medians and tertiles of the frequency of use in the specific study population<br><br>Each score component is allocated a maximum score of either one or two points, based on a supposition of the relative significance of the component for overall diet quality	The HFII final score is calculated as the sum of the 11 components (final score range is 0 to 17)	Nutrient intakes, component independence, construct validity, reproducibility, sociodemographic factors, body mass index, smoking and physical activity<br><br>All nutrient and (aligning with recommendations) showed a statistically significant association with an increased score. Energy intake was not significantly associated with scores<br><br>All components independently contributed to total score but for high-fiber grains and vegetables independency may have been weak<br><br>Factor analysis for the components revealed three distinct factors that explained most of the variation (59 %) within the score. This supports that the HFII measured meaningful dimensions of diet<br><br>Weighted Kappa coefficients between suggested moderate to substantial agreement<br><br>Years of education and higher physical activity was significantly associated with higher scores, while higher BMI and smoking was associated with lower scores

	Development				Scoring information		Evaluation
	Country of origin	Population	Dietary intake assessment	Evidence base used for DQI development	Scoring and weighting of components	Interpretation of score	
**Probability of Adequate Nutrient intake-based Diet quality index (PANDiet)**<br><br>Bianchi et al. (2016)[21]	Mainland France and the United States of America	French and US women of childbearing age and<br><br>pregnant women participating in the French Nutrition and Health Survey (Etude Nationale<br><br>Nutrition Santé—ENNS, 2006–2007) (N=344), and in the National Health Administration and Nutrition<br><br>Examination Survey (NHANES, 2009–2010) (N=563)	For the ENNS, dietary data were collected using three 24-hour recalls (including<br><br>a weekend day) randomly selected within a 2-week period. For the NHANES, two 24-hour recalls (week or weekend days), were used to obtain dietary intake data	Based on the PANDiet[36], and adapted to reflect nutrient requirements for pregnancy. For US women, nutrient recommendations of the IOM[73,38] and the Academy of Nutrition and Dietetics (AND)[39] were used. For French women, nutrient recommendations the French Agency for Food,<br><br>Environmental and Occupational Health[40,41] were used	A score that results from the average of two sub-scores, the Adequacy sub-score (AS) and the Moderation sub-score (MS) is calculated. Each sub-score is composed of probabilities of adequacy for nutrients (French population: 27 for the AS and 7 for the MS), with a further 14 potential penalties for exceeding tolerable upper intake limits which are added to the MS; US population: 25 for the AS and 5 for the MS), with a further 12 potential penalties for exceeding tolerable upper intake limits which are added to the MS<br><br>The Adequacy sub-score is calculated as the average of the probability of adequacy for components for which the usual intake should be above a reference value, multiplied by 100<br><br>The Moderation sub-score is calculated as the average of the probability of adequacy for components for which the usual intake should not exceed a reference value and penalty values, multiplied by 100	A minimum score of 0 and a maximum sore of 100 can be obtained	Nutrient intakes, food groups, energy density of diet, plasma folate, alpha and beta-carotene concentrations and smoking<br><br>French implementation:<br><br>All component (nutrient) scores (aligning with recommendations) improved significantly as total score increased, except PUFAs, zinc, vitamins A and B12<br><br>Participants with a higher score had a significantly higher intake of dairy, fish, fruit and vegetables and lower intakes of cheese, eggs, meat, processed meat and pizza<br><br>Participants with a higher score were more likely to be non-smokers and to have a lower-energy-dense diet<br><br>Participants with a higher score were more likely to have higher plasma folate, alpha and beta-carotene concentrations
**Diet Quality Index Adapted for Pregnant Women (IQDAG)**<br><br>Crivellenti et al. (2018)[22]	Sao Paulo, Brazil	Dietary intake data was obtained from a previous cross-sectional study (N = 785) on adult pregnant women	Two non-consecutive 24-hour dietary recalls as well as validated 85-item FFQ were used to obtain dietary intake information from pregnant women in their second and third trimester	Recommendations of the Ministry of Health[42], as well as the Revised IQD-R for the Brazilian population[25], the HEIP-B[16] and the Dietary Guidelines<br><br>for the Brazilian Population[43]	Each component contributes 0 to 10 points of total score. Intermediate intakes are scored proportionately between 0 and 10 by multiplying the number of servings that were consumed by 10, after which the score is divided by the standard for maximum score<br><br>Ultra-processed foods are weighted double than other components	A minimum score of 0 and a maximum sore of 100 can be obtained	Nutrient intakes, food groups, sociodemographic factors, body mass index, smoking, hypertension and gestational diabetes diagnosis<br><br>Total energy percentage from carbohydrates and proteins and the intake of cholesterol, vitamins, and minerals were positively correlated with the score, while the percentage of

	Development				Scoring information		Evaluation
	Country of origin	Population	Dietary intake assessment	Evidence base used for DQI development	Scoring and weighting of components	Interpretation of score	
							energy from total fat, saturated fat and monounsaturated fat were negatively correlated with total score<br><br>All component scores were significantly associated with total score<br><br>Higher scores were significantly associated with higher average age, higher activity, use of dietary supplements, and normal weight (according to the gestational week)
**Prenatal Diet Quality Index (PDQI)**<br><br>Borge et al. (2019)[23]	Norway	Pregnant women across Norway were enlisted in the Norwegian Mother and Child Cohort Study (MoBa) (N = 27 529)	A validated FFQ was used to obtain dietary intake data from pregnant women	Based on the HEI[44] and adapted to reflect the updated Norwegian food-based dietary guidelines[45]	Each component contributes 0 to 10 points of total score (however, total fish and fatty fish each contribute 5 points). Intermediate intakes are scored proportionately between 0 and 10 by multiplying the number of portions consumed by 10, after which it is then divided by the cut-off value for maximum score<br><br>Diversity score is based on the diversity of foods within the following four food groups: grains, vegetables, fruits, and dairy foods. Meal pattern is scored based on three main meals with two snacks or four main meals and one snack	A minimum score of 0 and a maximum sore of 120 can be obtained	Reproducibility, nutrient intakes, energy intake, sociodemographic factors, depressive symptoms, smoking, child developmental outcomes<br><br>Reliability analysis showed a Cronbach's α of 0.66 for the<br><br>PDQI, which is similar to the original HEI<br><br>PDQI scores were correlated with energy-adjusted intakes of key nutrients in the FFQ in the expected direction (fiber, sugar and saturated fat), as well as with key nutrients such as protein, iron, zinc, and B vitamins<br><br>A low correlation between energy intake and final scores was found

	Development				Scoring information		Evaluation
	**Country of origin**	**Population**	**Dietary intake assessment**	**Evidence base used for DQI development**	**Scoring and weighting of components**	**Interpretation of score**	
							The standardized mean difference between groups, reported as Hedges' *g. A* large effect size was found for education and age; a medium effect size was found for smoking, and a small-to- medium effect size was found for depressive symptoms

FFQ: food frequency questionnaire; DGAs: Dietary Guidelines for Americans; FGP: food guide pyramid; HEI: Healthy Eating Index; GDM: gestational diabetes mellitus; NNR: Nordic Nutrition Recommendations; ENNS: Etude Nationale Nutrition Santé; IOM: Institute of Medicine; AS: adequacy sub-score: MS: moderation sub-score; AND: Academy of Nutrition and Dietetics;
