# Supplementary material for: Diet quality indexes for use during pregnancy: a scoping review
Source: Nutr Rev. 2023 Oct 27;82(11):1622–30. doi: 10.1093/nutrit/nuad138 (PMC11464799; doi:10.1093/nutrit/nuad138)
Supplement: nuad138_Supplementary_Data [file nuad138_supplementary_data.zip › nuad138_Supplementary_Data/Table 2.pdf]

Table 1 Summary of components used in the current DQIs for pregnancy

	**Diet Quality Index for Pregnancy (DQIP)** Bodnar & Siega-Riz (2002)[13]	**Alternate Healthy Eating Index for Pregnancy (AHEI-P)** Rifas-Shiman et al. (2009)[14]	**Mediterranean Diet Score–Pregnancy (MDS-P)** Mariscal-Arcas et al. (2009)[15]	**Healthy Eating Index for Brazilian Pregnancy (HEIP-B)** Melere et al. (2013)[16]	**Diet Quality Index for Pregnancy (Canada) (DQI-Pc)** Nash et al. (2013)[17]	**Healthy Eating Index for pregnant women in Singapore (HEI-SGP)** Han et al. (2015)[18]	**Dietary Assessment Tool (DAT)** Mullaney et al. (2016)[19]	**Healthy Food Intake Index (HFII)** Meinilä et al. (2016)[20]	**Probability of Adequate Nutrient intake-based Diet quality index (PANDiet)#** Bianchi et al. (2016)[21]	**Diet Quality Index Adapted for Pregnant Women (IQDAG)** Crivellenti et al. (2018)[22]	**Prenatal Diet quality Index (PDQI)** Borge et al. (2019)[23]	**Use**
	**Use of components and references used for ideal scoring purposes**											
**Grains or cereals**	✓<br>FGP recommended servings (6-11) dependant on energy range[27]		✓<br>Above median intake		✓<br>CFG recommended servings (7)[32]		✓<br>No. of days per week with high fibre breakfast cereal					4/11
**Wholegrains**						✓<br>SDG for pregnant women recommended servings (≥1.30/1000 kcal)[34]		✓<br>FNR recommendations (≥3 x / d)[46]			✓<br>NDG (70g / d)[45]	3/11
**Rice and starchy alternatives**						✓<br>SDG for pregnant women recommended servings (≥2.60/1000 kcal)[34]	✓<br>Type of bread eaten (wholemeal/white/pitta) Serving size of cooked potatoes/rice/pasta at main meal					2/11
**Vegetables**	✓<br>FGP recommended servings (3-5) dependant on energy range[27]	✓<br>FGP recommended servings (upper range of 5)[27]	✓<br>Above median intake	✓<br>BDG recommended servings (≥3.5)[47]		✓<br>SDG for pregnant women recommended servings (≥1.30/1000 kcal)[34]		✓<br>FNR recommendations (> 2 / d)[46]		✓<br>DGB recommended servings (≥1.5/1000 kcal)[48]	✓<br>NDG (≥250 g / d)[45]	8/11
**Dark green leafy and orange vegetables**						✓<br>SDG for pregnant women recommended servings (≥0.21/1000 kcal)[34]						1/11

	**Diet Quality Index for Pregnancy (DQIP)** Bodnar & Siega-Riz (2002)[13]	**Alternate Healthy Eating Index for Pregnancy (AHEI-P)** Rifas-Shiman et al. (2009)[14]	**Mediterranean Diet Score–Pregnancy (MDS-P)** Mariscal-Arcas et al. (2009)[15]	**Healthy Eating Index for Brazilian Pregnancy (HEIP-B)** Melere et al. (2013)[16]	**Diet Quality Index for Pregnancy (Canada) (DQI-Pc)** Nash et al. (2013)[17]	**Healthy Eating Index for pregnant women in Singapore (HEI-SGP)** Han et al. (2015)[18]	**Dietary Assessment Tool (DAT)** Mullaney et al. (2016)[19]	**Healthy Food Intake Index (HFII)** Meinilä et al. (2016)[20]	**Probability of Adequate Nutrient intake-based Diet quality index (PANDiet)[#]** Bianchi et al. (2016)[21]	**Diet Quality Index Adapted for Pregnant Women (IQDAG)** Crivellenti et al. (2018)[22]	**Prenatal Diet quality Index (PDQI)** Borge et al. (2019)[23]	**Use**
**Fruit**	✓ FGP recommended servings (2-4) dependant on energy range[27]	✓ FGP recommended servings (upper range of 4)[27]		✓ BDG recommended servings (≥3.5)[47]		✓ SDG for pregnant women recommended servings (≥0.87/1000 kcal)[34]		✓ FNR recommendations (≥ 1 / d)[46]				5/11
**Whole/fresh fruit**						✓ SDG for pregnant women recommended servings (≥0.43/1000 kcal)[34]				✓ DGB recommended servings (≥1.5/1000 kcal)[48]	✓ NDG (≥250 g/ d)[45]	3/11
**Fruits and nuts combined**			✓ Above median intake									1/11
**Fruit and vegetables combined**					✓ CFG recommended servings (8)[32]		✓ No. of pieces of fruit/raw vegetables per day No. of servings of cooked vegetables or salad per day					2/11
**Beans/pulses /legumes**			✓ Above median intake	✓ BDG recommended servings (≥1.2)[47]						✓ DGB recommended servings (≥0.5/1000 kcal)[48]		3/11
**Dairy**			✓ Below median intake		cheese per week	✓ SDG for pregnant women recommended servings (≥0.43/1000 kcal)[34]	✓ Type of milk used (full fat/low fat/low fat fortified) Amount of milk per day Amount of	✓ ≤1% fat is used[49]			✓ NDG (3 servings / day)[45]	5/11

	**Diet Quality Index for Pregnancy (DQIP)** Bodnar & Siega-Riz (2002)[13]	**Alternate Healthy Eating Index for Pregnancy (AHEI-P)** Rifas-Shiman et al. (2009)[14]	**Mediterranean Diet Score–Pregnancy (MDS-P)** Mariscal-Arcas et al. (2009)[15]	**Healthy Eating Index for Brazilian Pregnancy (HEIP-B)** Melere et al. (2013)[16]	**Diet Quality Index for Pregnancy (Canada) (DQI-Pc)** Nash et al. (2013)[17]	**Healthy Eating Index for pregnant women in Singapore (HEI-SGP)** Han et al. (2015)[18]	**Dietary Assessment Tool (DAT)** Mullaney et al. (2016)[19]	**Healthy Food Intake Index (HFII)** Meinilä et al. (2016)[20]	**Probability of Adequate Nutrient intake-based Diet quality index (PANDiet)#** Bianchi et al. (2016)[21]	**Diet Quality Index Adapted for Pregnant Women (IQDAG)** Crivellenti et al. (2018)[22]	**Prenatal Diet quality Index (PDQI)** Borge et al. (2019)[23]	**Use**
**Cheese**								✓ ≤17% fat is used[49]				1/11
**Total protein foods**						✓ SDG for pregnant women recommended servings (≥1.08/1000 kcal)[34]						1/11
**Meat**			✓ Below median intake				✓ No. of days with processed red meats at the main meal per week<br>Serving size of meat/chicken/fish at the main meal<br>Usual cooking method for meat, poultry or fish					1/11
**Red meat**											✓ NDG (≤500 g / wk)[45]	2/11
**Fish**			✓ Above median intake					✓ FNR recommendations (≥ 1 / d)[46]			✓ NDG (300-450 g / wk)[45]	3/11
**Fatty fish**							✓ No. of servings of fresh or tinned oily fish per week				✓ NDG (200-450 g / wk)[45]	2/11
**White meat to red meat ratio**		✓ Based on AHEI recommendations (≥4:1)[50]		✓ Based on AHEI recommendations (≥4:1)[50]								2/11
**Fibre**		✓ Reference not stated (≥25 g)		✓ Reference not stated (≥25 g)					✓ IOM AI (14 g / 1000 kcal)[3]	✓ IOM AI (≥28 g)[51]		4/11

	**Diet Quality Index for Pregnancy (DQIP)** Bodnar & Siega-Riz (2002)[13]	**Alternate Healthy Eating Index for Pregnancy (AHEI-P)** Rifas-Shiman et al. (2009)[14]	**Mediterranean Diet Score–Pregnancy (MDS-P)** Mariscal-Arcas et al. (2009)[15]	**Healthy Eating Index for Brazilian Pregnancy (HEIP-B)** Melere et al. (2013)[16]	**Diet Quality Index for Pregnancy (Canada) (DQI-Pc)** Nash et al. (2013)[17]	**Healthy Eating Index for pregnant women in Singapore (HEI-SGP)** Han et al. (2015)[18]	**Dietary Assessment Tool (DAT)** Mullaney et al. (2016)[19]	**Healthy Food Intake Index (HFII)** Meinilä et al. (2016)[20]	**Probability of Adequate Nutrient intake-based Diet quality index (PANDiet)[#]** Bianchi et al. (2016)[21]	**Diet Quality Index Adapted for Pregnant Women (IQDAG)** Crivellenti et al. (2018)[22]	**Prenatal Diet quality Index (PDQI)** Borge et al. (2019)[23]	**Use**
**Folate**	✓<br>IOM RDA (600 ug DFE)[52]	✓<br>IOM RDA (600 ug DFE)[52]	✓<br>Spanish RDI (600 ug DFE)[53]	✓<br>IOM RDA (≥600 ug DFE)[52]	✓<br>IOM EAR (520 ug DFE)[52]				✓<br>IOM EAR (520 ug DFE)[52]	✓<br>IOM EAR (≥520 ug DFE)[52]		7/11
**Iron**	✓<br>IOM RDA (27 mg)[38]	✓<br>IOM RDA (27 mg)[38]	✓<br>Spanish RDI (18 mg)[53]	✓<br>IOM RDA (≥27 mg)[38]	✓<br>IOM EAR (22 mg)[38]				✓<br>IOM EAR (22 mg)[38]	✓<br>IOM EAR (≥22 mg)[38]		7/11
**Calcium**	✓<br>IOM AI for age (1000/1300 mg)[54]	✓<br>Reference not stated (1200 mg)	✓<br>Spanish RDI (1400 mg)[53]	✓<br>IOM AI for age (≥1000/1300 mg)[54]	✓<br>IOM AI for age (1000 mg)[54]				✓<br>IOM EAR for age (800 mg)[54]	✓<br>IOM EAR for age (800 mg)[54]		7/11
**Total fat**	✓<br>DGA recommendation of ≤30 %TE[26]				✓<br>CFG recommendation of 20 - 35 %TE[32]	✓<br>SDG recommendation of ≤30 %TE[34]			✓<br>IOM recommendation of 20 - 35 %TE[3]			4/11
**SFA**						✓<br>SDG recommendation of ≤10 %TE[34]			✓<br>IOM recommendation of <10 %TE[3]		✓<br>NDG (≤10% TE)[45]	3/11
**Ratio of PUFA: SFA**		✓<br>Based on AHEI recommendations (>1)[50]		✓<br>Based on AHEI recommendations (≥4:1)[50]								2/11
**Ratio of MUFA: SFA**			✓<br>Above median intake									1/11
**Trans fat**		✓<br>Based on AHEI recommendations (≤0.5% TE)[50]		✓<br>Reference not stated (0 g)							✓<br>NDG (≤1% TE)[45]	3/11
**Cholesterol**									✓<br>IOM recommendation of < 300 mg[3]			1/11
**Omega-3 FA / EPA + DHA**									✓<br>AND recommendation of > 385 mg DHA + EPA[39]	✓<br>IOM AI (≥1.4g omega-3 FA)[51]		2/11

	**Diet Quality Index for Pregnancy (DQIP)** Bodnar & Siega-Riz (2002)[13]	**Alternate Healthy Eating Index for Pregnancy (AHEI-P)** Rifas-Shiman et al. (2009)[14]	**Mediterranean Diet Score–Pregnancy (MDS-P)** Mariscal-Arcas et al. (2009)[15]	**Healthy Eating Index for Brazilian Pregnancy (HEIP-B)** Melere et al. (2013)[16]	**Diet Quality Index for Pregnancy (Canada) (DQI-Pc)** Nash et al. (2013)[17]	**Healthy Eating Index for pregnant women in Singapore (HEI-SGP)** Han et al. (2015)[18]	**Dietary Assessment Tool (DAT)** Mullaney et al. (2016)[19]	**Healthy Food Intake Index (HFII)** Meinilä et al. (2016)[20]	**Probability of Adequate Nutrient intake-based Diet quality index (PANDiet)[#]** Bianchi et al. (2016)[21]	**Diet Quality Index Adapted for Pregnant Women (IQDAG)** Crivellenti et al. (2018)[22]	**Prenatal Diet quality Index (PDQI)** Borge et al. (2019)[23]	**Use**
**Cooking fat**								✓ Mostly vegetable oils[49]				1/11
**Fat spreads**								✓ Mostly oil-based[49]				1/11
**Ultra-processed foods (total)**										✓ Cut-off points are based on the 16th and 85th percentile of the distribution curve of the intake of UPF by the study population[55]		1/11
**Salt / Sodium**									✓ IOM recommendation of < 2400 mg sodium[3]		✓ NDG(≤6 g salt / d)[45]	2/11
**Added sugar/sugary foods and drinks**							✓ No. of sweet cakes/biscuits per week No. of teaspoons of sugar, honey or jam per day No. of sugar-sweetened fizzy drinks per week				✓ NDG (≤10 % TE added sugar)[45]	2/11
**SSBs**								✓ Based on median intake (<1 x / wk)				1/11
**Fast foods**								✓ Based on median intake (<1 x / wk)				1/11
**Snacks/fatty foods**							✓ No. of servings of chips per week	✓ Based on tertiles (≤4 x / wk)				2/11

	**Diet Quality Index for Pregnancy (DQIP)** Bodnar & Siega-Riz (2002)[13]	**Alternate Healthy Eating Index for Pregnancy (AHEI-P)** Rifas-Shiman et al. (2009)[14]	**Mediterranean Diet Score–Pregnancy (MDS-P)** Mariscal-Arcas et al. (2009)[15]	**Healthy Eating Index for Brazilian Pregnancy (HEIP-B)** Melere et al. (2013)[16]	**Diet Quality Index for Pregnancy (Canada) (DQI-Pc)** Nash et al. (2013)[17]	**Healthy Eating Index for pregnant women in Singapore (HEI-SGP)** Han et al. (2015)[18]	**Dietary Assessment Tool (DAT)** Mullaney et al. (2016)[19]	**Healthy Food Intake Index (HFII)** Meinilä et al. (2016)[20]	**Probability of Adequate Nutrient intake-based Diet quality index (PANDiet)[#]** Bianchi et al. (2016)[21]	**Diet Quality Index Adapted for Pregnant Women (IQDAG)** Crivellenti et al. (2018)[22]	**Prenatal Diet quality Index (PDQI)** Borge et al. (2019)[23]	**Use**
							No. of packets of crisps/savoury snacks per week					
**Alcohol**							✓ Usual no. of units per week					1/11
**Antenatal supplement use**						✓ SDG for pregnant women recommendations for supplement use (contain iron, folate and calcium)[34]	✓ No. of times per week taking a vitamin D supplement No. of times per week taking a multivitamin supplement No. of times per week taking an Omega-3 supplement					2/11
**Meal pattern**	✓ IOM recommendation (three meals and at least two snacks)[56]										✓ IOM recommendation (three meals and at least two snacks)[56]	2/11
**Dietary diversity**											✓ Diversity score is based on diversity of foods within 4 major food groups eaten daily (grains, vegetables, fruits, and dairy foods)[57]	1/11
**Vitamins A, B1, B2, B3, B6, B12, C, D, E, calcium, magnesium, phosphorus,**									✓ See Bianchi et al (2016) for scoring information[21]			1/11

	**Diet Quality Index for Pregnancy (DQIP)** Bodnar & Siega-Riz (2002)[13]	**Alternate Healthy Eating Index for Pregnancy (AHEI-P)** Rifas-Shiman et al. (2009)[14]	**Mediterranean Diet Score–Pregnancy (MDS-P)** Mariscal-Arcas et al. (2009)[15]	**Healthy Eating Index for Brazilian Pregnancy (HEIP-B)** Melere et al. (2013)[16]	**Diet Quality Index for Pregnancy (Canada) (DQI-Pc)** Nash et al. (2013)[17]	**Healthy Eating Index for pregnant women in Singapore (HEI-SGP)** Han et al. (2015)[18]	**Dietary Assessment Tool (DAT)** Mullaney et al. (2016)[19]	**Healthy Food Intake Index (HFII)** Meinilä et al. (2016)[20]	**Probability of Adequate Nutrient intake-based Diet quality index (PANDiet)#** Bianchi et al. (2016)[21]	**Diet Quality Index Adapted for Pregnant Women (IQDAG)** Crivellenti et al. (2018)[22]	**Prenatal Diet quality Index (PDQI)** Borge et al. (2019)[23]	**Use**
**potassium, selenium, zinc**												
**Protein, LA, ALA, DHA, total carbohydrate**									✓ See Bianchi et al (2016) for scoring information[21]			1/11
	**Additional notes**											
**Intake from micronutrient supplements is considered**			✓							✓	✓	3/11
**Energy density considered**	✓					✓				✓		3/11

FGP: food guide pyramid; CFG: Canadian Food Guide; SDG: Singapore Dietary Guidelines; FNR: Finnish Nutrition Recommendations; NDG: Norwegian Dietary Guidelines; BDG: Brazillian Dietary Guidelines; DGB: Dietary Guidelines for Brazillians; AHEI: Alternate Healthy Eating Index; IOM: Institute of Medicine; RDA: recommended dietary allowance; EAR estimated average requirement; AI: adequate intake; DGA: Dietary Guidelines for Americans; #Only US nutrient recommendations indicated
